# PB.07: Why are some fibroadenomas stiff using shear wave elastography?

**DOI:** 10.1186/bcr3509

**Published:** 2013-11-08

**Authors:** M Elseedawy, P Whelehan, S Vinnicombe, K Thomson, A Evans

**Affiliations:** 1 Dundee Cancer Centre, Dundee, UK; 2 Ninewells Hospital, Dundee, UK; 3 University of Dundee, UK

## Introduction

Shear wave elastography (SWE) is a promising modality for differentiating benign from malignant breast masses. A proportion of fibroadenomas are stiff (mean stiffness >50 kPa), resulting in false positive SWE findings. The aim of this study was to identify which features of fibroadenomas are associated with false positive SWE findings.

## Methods

A total of 151 patients with histologically confirmed fibroadenomata were identified from a prospective database, from a single breast unit. The following features were assessed by a single observer who was unaware of the SWE findings: Patient age, greyscale ultrasound lesion diameter (<15 mm or ≥15 mm), distance from the lesion to skin, composition of surrounding tissue (fatty, mixed or dense) and source of referral (screening or symptomatic). Statistical analysis was carried out using the chi-square test.

## Results

A statistically significant association was found between greyscale ultrasound lesion size and lesional stiffness. Twenty-nine of 70 (41%) lesions ≥15 mm were stiff, versus 10 of 81 (12%) <15 mm, *P *= 0.001. Patient age, distance from the lesion to skin, makeup of surrounding tissue and source were not significantly associated with stiffness.

## Conclusion

Fibroadenomas giving false positive SWE results tend to be larger in size than these that do not. More compression of adjacent normal tissue is assumed to be the cause of our findings. As previous studies have shown that large cancers tend to be stiffer than smaller cancers, it may be appropriate to vary the quantitative cutoff value used for benign/malignant differentiation in SWE according to lesion size.

